# Dutch Primary Schoolchildren’s Perspectives of Activity-Friendly School Playgrounds: A Participatory Study

**DOI:** 10.3390/ijerph13060526

**Published:** 2016-05-24

**Authors:** Helena Elisabeth (Elsje) Caro, Teatske Maria Altenburg, Christine Dedding, Mai Jeanette Maidy Chinapaw

**Affiliations:** 1 Department of Public and Occupational Health, VU University Medical Center, EMGO Institute for Health and Care Research, Amsterdam 1081 BT, The Netherlands; he.caro@vumc.nl (H.E.C.); m.chinapaw@vumc.nl (M.J.M.C.); 2 Athena Institute for Research on Innovation and Communication in Health and Life Sciences, VU University, Amsterdam 1081 HV, The Netherlands; c.dedding@vu.nl

**Keywords:** physical activity, active play, activity-friendliness, child researcher, participation

## Abstract

School playgrounds are important physical activity (PA) environments for children, yet only a small number of children reaches the target of 40% of moderate-to-vigorous PA time during recess. The aim of this study was to explore children’s perspectives (*i.e.*, child-identified determinants) of activity-friendly school playgrounds. We conducted participatory research with children as co-researchers, framed as a project to give children the opportunity to discuss their views and ideas about their school playgrounds. At three schools, six children (9–12 years old) met over five to seven group meetings. Data analysis included children’s conclusions obtained during the project and the researcher’s analysis of written reports of all meetings. Children indicated a strong desire for fun and active play, with physical playground characteristics and safety, rules and supervision, peer-interactions, and variation in equipment/games as important determinants. Our results indicate that improving activity-friendliness of playgrounds requires an integrated and multi-faceted approach. It also indicates that children, as primary users, are able to identify barriers for active play that are easily overlooked, unknown or differently perceived by adults. Hence, we believe that structural involvement of children in designing, developing and improving playgrounds may increase children’s’ active play and consequently PA levels during recess.

## 1. Introduction

The beneficial health effects of physical activity (PA) are well established [1]. However, a recent European study using accelerometer-based PA levels in 10–12 years old children showed that only 5% of the girls and 17% of the boys meet the PA recommendation for 5–17 years old children of at least 60 min of daily moderate-to-vigorous physical activity (MVPA) [1,2,3]. Promoting PA among schoolchildren is thus of significant importance, because PA behaviour also tracks from childhood into adulthood [4,5]. 

Schools are important settings to encourage children to be physically active [6], since almost all children can be reached, irrespective of socio-economic or ethnic background, and children spend a large majority of their day at school. Recess time in the playground offers an important daily opportunity to engage in MVPA, thereby contributing to the above-mentioned PA recommendation [1,3,7]. A target of 40% of MVPA time during recess has been suggested as feasible [8], though only 12% to 44% of 6–11 year old Dutch girls and boys, respectively, reach this percentage [7]. Previous studies suggest that outdoor time is associated with more PA [9] and that recess time at primary schools has the potential to contribute to up to one-third of the recommended MVPA a day, especially when children have a continuous schedule (*i.e.*, stay at school throughout the day including lunchtime) [10]. Dutch children are increasingly on a continuous schedule, which may provide more recess time during the day. Importantly, children may also use the school playground outside school hours [7]. 

The development of playgrounds that stimulate children to be physically active may be an effective strategy to enhance children’s PA levels. Two systematic reviews on the role of playground characteristics in playground PA showed that experimental studies gave moderate evidence for an effect of multi-component interventions on playground PA levels of primary schoolchildren [11]. Inconclusive evidence was found for the beneficial effects of providing equipment, using playground markings, and allocating play space for team games [11]. Observational studies indicated a positive association of decreased playground density (m^2^/child), fixed (unspecified) [11] and loose (unspecified and balls) equipment with PA levels [11,12]. No associations were reported for playground surface type, increased recess duration, playground size, safety and quality [11]. The ecological approach to PA suggests that the social and policy school domains are important environmental determinants of children’s playground PA [13,14]. The activity-friendliness of playgrounds can be considered as an interplay between multiple environmental characteristics.

Overall, research regarding children’s perspectives of the activity-friendliness of playgrounds is scarce. This is surprising, since increasing understanding of the primary users’ perspectives of playgrounds through qualitative research could result in new insights regarding environmental influences on playground PA [15,16], including general play spaces [17]. Previous qualitative studies indicate that children consider physical characteristics, such as the presence and access to equipment and playground aesthetics [18,19,20,21,22], social characteristics like bullying and conflicts [18,19,20], school policy [19] and weather [18,19,20] as important characteristics. 

To date, the use of a participatory approach, *i.e.*, in which academic researchers and children actively collaborate in one or more phases of the research process [23], is not common in PA research. There is increasing recognition that compared to traditional qualitative research this type of research can enhance understanding of how children view and experience their living world [16,23,24]. In participatory child research, children are acknowledged as experts in their own lives and capable of participating in research about matters that affect them [16,23,25]. Through shared decision-making they are given the opportunity to express their perspectives throughout the research process and outcomes [26,27,28]. Previous participatory studies demonstrated that this principle and collaborating over time enable the development of a relationship of trust between academic researchers and the children, ultimately revealing insider insights that are helpful in gaining more in-depth understanding of the problem under study, in creating interventions that are accepted by children, and in improving the utilization and dissemination of research findings [23,27,29,30]. Finally, participatory research enables mutual learning between researcher and children and often aspires action and change in order to improve the lives of children [27]. 

The aim of this study was to explore child-identified determinants of activity-friendly school playgrounds. We used a participatory research approach, *i.e.*, collaborating with children as co-researchers, and focused on children in the last years of primary school (aged 9–12 years). 

## 2. Materials and Methods

### 2.1. Ethics

The study was reviewed by the VU University Medical Ethical Committee (reference number: 2013/278). After obtaining permission from the school head, both participating children and their parents received an information letter on the goals and procedures of the study, and both were asked to provide written informed consent. 

### 2.2. Study Design

This study used a participatory research design that was framed as a project giving primary schoolchildren the opportunity to discuss their views and ideas about their school playground and PA. Children were invited as co-researchers and could actively influence the form and content of the research process as well as the dissemination of their findings. Children indicated which playground topics they wanted to examine and how they wanted to do this. Children summarized their findings during this process, thereby actively contributing to the data analysis. 

Schools were screened for being open and having an interest in using the children’s suggestions to improve the school playground. However, this study was not specifically set up to undertake action on this as part of the research (*i.e.*, participatory *action* research). Nevertheless, several methods were used to communicate the study findings to those who were able to create positive changes [31]. First, children were encouraged to present their findings to those who they considered “influential”. Second, the researcher summarized children’s findings in a factsheet that was reviewed by the children and subsequently communicated to the school staff. Finally, the researcher presented and discussed the children’s findings from all three schools to a mixed audience, which included two children, one parent, one teacher and three public health professionals. 

### 2.3. Participants

Thirty-four primary schools in and around Amsterdam were invited to participate, through emails, follow-up phone calls and the professional networks of the researchers (*n* = 22) and the Public Health Service of Amsterdam (*n* = 12). Inclusion criteria included: that the schools were open to and interested in ideas from their pupils about their playground as well as implementing some of these ideas. Schools were screened for these criteria through an interview with the teacher who was the primary contact person for this project. To ensure variation in socioeconomic status of the school neighbourhood, socioeconomic status scores for 2010 were obtained from The Netherlands Institute for Social Research [32]. This status score indicates the social status of people within a certain postal code area by combining data on income level, level of education and employment status [32]. Tertile cut-off values based on all status scores in the Netherlands were used to distinguish between low, medium and high socioeconomic status. The project was conducted in December 2013–February 2014 (School 1), February–March 2014 (School 2) and May–June 2014 (School 3).

Three eligible schools in Amsterdam agreed to participate. Table 1 presents the neighbourhood, school, playground, recess and participant characteristics. At each school a group of six boys and girls from the highest two grades (Dutch educational system: group 7/8) was formed. Limiting the group to six participants allowed for lively but manageable discussions [33,34]. At School 1, the teacher introduced the project and selected children based on their motivation to participate, ability to express themselves and whether the project would fit within their personal learning schedule. At Schools 2 and 3, the researcher briefly explained the project to the participating classes, after which the children could express their willingness to participate. All children in School 2 and all but three children in School 3 were willing to participate. Of those who were willing to and had permission from their parents to participate, six were randomly selected by means of drawing lots. 

### 2.4. Procedures

As the research process in participatory research is shaped by the participants as part of the research itself, detailed project activities were unknown before the start of the project. A general outline of the participatory group meetings as well as a detailed description on how the project was developed in each school is presented below.

#### 2.4.1. Participatory Group Meetings: General Outline

In each school, the children met with the researcher in five to seven group meetings of approximately 1–1^½^ h per meeting. The number of meetings depended on the progress (e.g., whether new topics emerged from discussions) and the available time for the project. Meetings were held during school hours in a room located within the building and in the school playground. To help to create an informal environment, the room was generally not a traditional classroom (e.g., the physical education room or the auditorium) [16]. 

The researcher introduced the overall subject of the project in a neutral way by asking children to explore their ideas regarding playgrounds that were suitable for PA (which was described as “moving”). During the meetings multiple playground topics were discussed, often introduced by the children and sometimes by the researcher. The researcher facilitated the group process, among others by sharing knowledge and skills, providing materials, managing discussions, paying attention to how to work in a pleasant and “professional” way and keeping track of time. Children chose the research techniques (see “Results”) helping them to express their ideas [16,28,37]. Depending on the group dynamics, the researcher actively brought in techniques to stimulate an active and focused atmosphere. Time was reserved to prepare a final product in which the group summarized their findings. 

Children were encouraged to propose home assignments that could serve as preparation for the next meeting. They received a journal for personal notes, home assignments and copies of group work such as meeting summaries. In addition, the journal contained a short questionnaire with items on gender, age, motivation to participate and initial opinion about the playground. At the end of the project, children completed an evaluation questionnaire (adapted from existing questionnaires [34,38]) with items investigating their overall feeling about the project, their role as co-researcher, the performance of the academic researcher and the results of the project. Before the start of the project at School 1, the researcher pre-tested this questionnaire with two primary schoolchildren ages 10 and 12 years old, to gain insight into the comprehensibility of the items in terms of terminology, as well as appropriateness of the reading level and ambiguity.

At Schools 2 and 3, two academic researchers participated in the group meetings, thereby reducing observer bias (*i.e.*, investigator triangulation). At School 1, only one academic researcher participated. After each group meeting, the researchers reviewed the content and the group process, including their role as facilitators, to identify issues that needed further investigation and to consider possible facilitation strategies that could be used during the next meeting. All group meetings were audiotaped and together with observations summarized in detailed written meeting reports.

#### 2.4.2. Participatory Group Meetings: Project Development per School

At School 1, the project was started with brainstorming about the concept “school playground”, followed by formulating questions that could be central in future meetings. The facilitating researcher suggested five research questions such as: what are positive and negative aspects of the playground? and how can we improve our playground? Children discussed the appropriateness of these questions and approved all questions, though two required editing. Children also formulated two additional questions: how is our playground? and are we satisfied with our current playground? Children chose different participatory activities to answer the questions, including taking pictures, making drawings (once with input from their classmates) and keeping a playground diary. Additionally, children chose activities from a list of suggestions of the researcher, including an exploratory walk across the school playground, role playing and answering question cards. When discussing to whom and how to present their findings, children decided to summarize and present their findings in a PowerPoint presentation to their school head, members of the playground discussion group (teacher, architect of the school building, parents) and their playground supervisors. These people were invited to the presentation (and were all present), as the children believed they were in the position of actually changing the playground. A boy initially hesitated to invite those playground supervisors that were not friendly in his eyes. The group replied as follows. Girl: “*Yes, but it’s especially useful if they hear that*”. Boy 2: “*That they think, like, “Hey, we should behave more normally”*”. One or two children were absent during a few meetings due to illness, vacation and once because a child gave priority to a special lesson that was given simultaneously. 

The researcher observed that the approach of separating playground topics in different questions was sometimes perceived as artificial and redundant to children, because to them the topics were closely interrelated and therefore often discussed simultaneously. In addition, postponing the preparation of the final product to the end of the project resulted in repetition of discussions as well. Therefore, to optimize children’s research process, children at Schools 2 and 3 worked on the final product from the start of the project, as a vehicle guiding the process. 

At School 2, the first group meeting was largely dedicated to formulating a project plan. The researcher asked the children to suggest important playground issues and to whom and how the children wanted to present their findings at the end of the project. The children decided focusing their research on what they called “the small playground” (Table 1, section I) that was mainly used by the younger children, instead of “the large playground” (Table 1, section II) that was mainly used by the older children. As the small playground was the property of the school and the large playground was under the control of the municipality, the children evaluated the chance to actually change the playground higher for the smaller playground. The children chose to explore issues “on the spot” by means of group discussions in the playground. In response, one girl stated: “*Miss, this really helped me come up with ideas because we were outside (in the playground)*”. Ideas were subsequently reflected upon and ordered by the children following questions of the researcher. Hereafter, children used the meetings to prepare, rehearse and present a PowerPoint presentation about their ideas to a self-selected audience. The audience consisted of their teacher, classmates and children of the youngest groups (aged 4–6 years). The latter were invited because the participating children considered it a right of the younger children—as the primary users of the small playground—to hear about and give feedback on their ideas. The school head and the children’s counsellor were invited as well, but unfortunately were unable to come. Upon a suggestion of one of the children the researcher informed the school head by mailing her the presentation. For the most part, all children were present during the group meetings, except for one occasion when a boy was absent because of illness. 

At School 3, an open and child-led conversation about the playground developed during the first meeting. At the end, a girl suggested to visit the playground to see what could be changed. This visit was the major activity during the second meeting and resulted in children spontaneously interviewing each other. In addition, children in this school decided to develop and set out a small questionnaire to ask their classmates about the playground. After brainstorming about possible ways to summarize their findings, the children decided to shoot a self-made film about the multiple playground issues for the school head, their teacher and classmates. The children felt strongly that important considerations should be presented to the school head. They were also concerned about the opinions of their classmates. The film script was developed through improvisation. Importantly, during acting and playing in the playground, children uncovered new issues that were incorporated into the film. Children wanted to record the scenes themselves, though for practical reasons they sometimes handed over this task to the academic researcher. Because of the short time frame, the researcher edited the film. Children commented on preliminary versions of the film, leading them to adapt and add scenes until all approved a final version. The children presented the film to the audience, along with a short oral presentation.

#### 2.4.3. Informal Meetings

Besides the planned group meetings, the researcher had several informal communications with the children (e.g., joining the children and their classmates in the playground during a school recess and spontaneous conversations prior and after group meetings). At School 3, children decided to communicate through group emails and WhatsApp messages as well. These informal interactions helped the researcher to develop a relationship of trust with the children, to establish rapport and to gain a deeper understanding of the ideas that were discussed during the group meetings. Informal communications were not audiotaped but described later in written meeting reports.

### 2.5. Data Analysis

Data analysis went through several iterative stages and was partly integrated within the data collection phase. First, children were asked to draw conclusions during and at the end of the project (*i.e.*, when they prepared their final product). Second, the researchers identified and interpreted main playground issues in the course of the project and these were communicated to the children for feedback and further investigation during subsequent meetings. Third, all written meeting reports were carefully read by the researcher and analyzed using the qualitative data analysis software program ATLAS.ti 5.2 (Scientific Software Development GmbH, Berlin, Germany). Part of the reports was analyzed through open coding, in which the researcher identified text fragments corresponding to single playground subjects. These fragments were given a code, e.g., undamaged, clean. Then the reports were analyzed using a combination of open and axial coding, in which codes were redefined (*i.e.*, axial coding), new codes emerged (*i.e.*, open coding) and related codes were summarized into categories (*i.e.*, axial coding), e.g., state of maintenance. Using selective coding, these categories were then integrated into themes that reflected the core of our results, e.g., physical characteristics. Although the described process seems more or less linear, it was in fact iterative, creating a scheme of codes, categories and themes that reflected the content of all reports [39], thereby taking into account the perspectives of the children. The scheme was subsequently discussed and revised with the research team. Other materials created by children, such as photos, drawings and free notes, served as vehicles to stimulate thinking and discussion during the group meetings. 

## 3. Results

### 3.1. Activity Behaviour in the Playground: Fun Active Play

In talking about their playground behaviour and playground preferences, it appeared that most children have a strong wish to be physically active in the playground, e.g., to run, climb, jump, swing, slide, play soccer and do traditional and self-invented playground games. Illustrative is the contribution of two girls to a brainstorm explaining their wish to set up competitions. Girl 1: “*All competitions and so on. (…) For example, we can jump on a trampoline, then you have to try to jump really high for example. In the sandpit, with the sandpit as starting point, who can run the fastest and then you have to jump over the sandpit and then there are nets you have to go underneath*”. Girl 2: “*Yeah, breaking records*.” *(School 2)*.

Soccer and basketball were also referred to as “sports”. Some children expressed a desire to engage in more sedentary activities as well, including sitting, talking, reading, lying in the sun and playing with electronic devices. Children often referred to these sedentary play activities as “playing” as well. Some children explained that sedentary activities, including moping and messing around, resulted from a shortage in play opportunities or play ideas.

Children emphasized the experience of fun as an important aspect of play and frequently spoke about it as a condition for engaging in play activities. The following two accounts are illustrative. A girl explained that it is strange to ask children why they do certain activities in the playground: “*Well, I think* “*why*” *is a bit strange too. Because when you say:* “*Yes, I play tag. Why do you play tag? Because it’s fun.*” *But that goes for most things. (…) Why do you play soccer? Because it’s fun. But that applies for most things, just because it’s fun.*” *(School 1)*. A boy said: “*You‘ve got to have fun in the playground.*” *(School 1)*.

In line with this, children’s evaluations of their playground were in general related to its opportunities for “fun active play”. When these were perceived as sufficient, children described their playground as “enjoyable”, while limited opportunities resulted in evaluating the playground as “boring” or “stupid”: “*Normally it’s not that enjoyable, because there’s not a lot you can do here.*” *(boy, School 3)*. A girl explained: “*I want to have more fun in that playground, because it’s just really boring.*” (*School 2*). The major themes that children indicated as influencing their opportunities to participate in fun active play in the playground were summarized as: (1) physical characteristics; (2) physical safety; (3) rules and supervision; and (4) peers. 

#### 3.1.1. Physical Characteristics

As school playgrounds are foremost a physical setting, children had much to say about physical characteristics of their playground. Children’s discussions focused on play space, play equipment, state of maintenance and aesthetic elements.

##### Play Space: Size and Free Space

Children agreed that large playgrounds were preferred over small playgrounds. More room to play—especially to run—was one of the mentioned arguments. Children explained that large playgrounds are also attractive because they can harbour more fixed play equipment. Importantly, they experienced that fixed play equipment could also hinder their play opportunities, especially when the equipment is placed close together. This is because they limit the amount of available and uninterrupted free space that children need for specific play activities. For example, a girl explained: “*I think those walls get in the way a bit. When you want to run around, the walls are in the way all the time.*” *(School 1)*. The importance of free space was also illustrated by some children who expressed their irritation about space that was often obstructed by bikes: “*Near that container too, which is a very good place to hide, but there are bikes everywhere, so you can’t use that area.*” *(boy, School 3)*. Further, an overcrowded playground was described as limiting the available play space, because children are getting in the way.

##### Play Equipment: (Amount of) Fixed, Loose, Alternative, Variation, Accessibility, Level of Difficulty, Suitability and Quality

Children reported that both fixed and loose equipment have an important role in their playground play. Most children were enthusiastic about equipment such as climbing frames, swings, slides, tumble bars, soccer/basketball fields with goals, table tennis tables, trampolines and seesaws (fixed equipment), stilts, elastics, balls and accompanying materials such as rackets and bats (loose equipment). Besides equipment that is designed for play, also alternative play materials emerged from discussions, such as lampposts, stairs, fences, edges and nature. A girl explained how stairs were used for play: “*Then we go three steps up, four down and who makes a mistake is finished.*” *(School 1)*. Many felt that more equipment would improve the play opportunities and hence the fun factor of the playground. It was discussed that more of the same equipment enables more children to engage in a specific play activity, whereas more different kinds of equipment allows children to vary play activities. For example, a girl clarified: “*It’s all a bit of the same, there should be some different things.*” *(School 2)*. Moreover, a varied supply would better meet individual play preferences of children. As a girl explained about her wish to have tennis equipment: “*For example, some children don’t want to play basketball, don’t want to play soccer and then they can play tennis for example. And then you need a tennis ball and a racket for example.*” *(School 3)*.

Children were often strongly motivated to have playgrounds that are attractive for both younger and (themselves as) older children and emphasized that variation in play equipment is essential to achieve this. In response to the researcher’s question as to why a seesaw should be added after a girl had recently mentioned that seesaws are boring: “*It’s more for the little ones, they like it.*” *(School 2)*. In general, participating children criticized their current playground for the few opportunities for fun active play. Two—partly interrelated—motives underlay their criticism: lack of challenge and height aspects. First, the equipment should have a level of difficulty that fits their age so that playing with the equipment is exciting and not too easy. For example, the children preferred a mix of low and high climbing frames and stepping stones with less and more separation distance (suitable for both younger and older children). Second, some children experienced that they were too tall for some of the available equipment: “*Those tumble bars are higher, which makes tumbling easier.* *With these bars, your hair sweeps the ground.*” *(girl, School 1)*. Opinions on the existence of gender differences in equipment preferences were mixed. 

Conversations about play equipment did not only focus on the composition of the total of play equipment, but also concentrated on characteristics of individual play materials. Children talked about discomfort they experienced with some current equipment and made suggestions on how to improve equipment in the light of active play and fun. The following examples are illustrative. Some children felt uncomfortable about using the slide, because of experiencing static electricity shocks: “*I didn’t dare use the slide because of the shocks I get when I use it.*” *(girl, School 3)*. In addition, some explained that slides should be longer, “*Because you’re on the slide for longer, and because it’s faster then.*” * (girl, School 2)*. Many children expressed their enthusiasm about equipment in which more than one child can be involved at one time. For example, multiplayer swings and multiplayer slides were preferred over single ones. About balls, children reported that not every ball is fun to play with. Aspects such as hardness and whether a ball can bounce influence play opportunities. Equipment quality was considered important in view of the required resistance against damage.

Access to equipment emerged as an important issue from discussions. Children reported several factors that hindered access. First, when lots of children have recess simultaneously, most equipment is occupied and consequently children have to wait their turn. This was also experienced as a source for arguments between children. Some children reported that occupied equipment and space caused them to engage in less interesting active play or non-active play such as sitting and chatting. For example, during the playground walk, a girl explained while pointing to some children: “*You see, there are more who want to play soccer, but it’s not possible right now and they all sit at this moment.*” *(School 3)*. Second, the positioning of soccer goals underneath baskets results in inaccessible baskets: “*You can’t use that anyway, because everybody plays soccer there.*” *(boy, School 1)*. Third, loose play equipment that is the property of individual children instead of the school can result in reduced access, since non-owners have to ask and get permission before they can play with the equipment. Fourth, soccer schedules, indicating which group can use the soccer field on what day, resulted in reduced access to the game of soccer. Although children considered the use of a schedule fair—it guarantees that each group gets a turn—they were unsatisfied about the limited possibilities to play soccer. One child suggested breaking up the recess into two smaller ones per day, so that each group could have the field more often. Another introduced the idea to create a second soccer field. 

##### State of Maintenance

Children considered it important that their school playground was undamaged and clean. For example, broken or worn-out equipment and pavement should be repaired or replaced. Children of Schools 1 and 2 had a lot to say about playground hygiene. It was described that some places and equipment were dirty and stinking due to urine, vermin, mud, sand or moisture, and therefore could not be used with pleasure. A boy about the slide: “*It’s a natural toilet, a toilet for homeless people.*” *(School 2)*. Further, it was explained that a messy playground could result in getting messy yourself. While some did not mind, others did not appreciate that. Regular cleaning, for example by teachers, an external cleaning service, children or those who make it dirty, was mentioned as a strategy to improve hygiene. Another proposed solution included covering equipment such as the sandpit with sheets outside recess time. Other suggestions for the sandpit were to freshen the sand on a regular basis, to make it smaller or to remove it entirely. 

##### Aesthetic Elements

Many children identified a cosy and friendly appearance as an important quality of enjoyable playgrounds. Some children expressed dissatisfaction about the current grey look of their playground: “*It, like, gives off such a negative feeling.*” * (boy, School 1)*. Multicoloured playgrounds with natural or lively colours were proposed instead. A fenced playground was not always liked, since some children mentioned that it looked a little like a prison. In addition, not all children were convinced that their current fence was effective in combating the problems they mentioned as arguments for fencing (against vandalism, thieves and to prevent children from leaving the playground). Nature such as trees, bushes, flowers, plants and grass were valued for their beauty and the play opportunities they provide. It was explained that nature can for example be used for hide and seek games, climbing and rolling on the ground. Some children wanted to have a vegetable and fruit garden, e.g., “*Such a garden with soil and then plant stuff.*” *(girl, School 2)*. In addition, there was enthusiasm about the idea to have animals in the playground. Some children felt disappointed about the absence of urban wildlife: “*When your class arrives on the playground first, that moment when nobody’s been there yet, then you might see three pigeons there, but that’s all.*” *(girl, School 1)*. Some suggested to keep farm animals and pets in the playground. Though a boy commented that it would be difficult to guarantee care and money for them. 

#### 3.1.2. Physical Safety

Children were concerned about their own and other’s safety in the playground during recess, especially that of the younger ones. Children described that they become hurt due to accidental falling, slipping and colliding with one another and felt that several characteristics of the playground environment could influence the risk for injuries. Safety concerns included surface safety, equipment safety and safety of playground surroundings.

##### Surface Safety

Soft surface was preferred underneath play equipment to provide fall attenuation: “*Not as soft as a pillow or something similar, but somewhat softer than stone.*” *(girl, School 1)*. At the same time, many children experienced that the soft tiles currently present in their playground were hardly softer than normal paving stones. At open areas, simple paving stones were accepted. At School 3, some places had sand and gravel on top of a hard surface. This combination was strongly disliked for safety reasons, as illustrated by the following account: “*Well look, you have, say, that gravel, and when you fall, say, little pieces of gravel cut into your hand.*” *(girl, School 3)*. In addition, broken, loose and wobbling tiles were considered dangerous because they cause children to stumble and fall on the ground. One child reported to have slipped on leaves. 

##### Equipment Safety

Children discussed several experiences and ideas in which they related the design of playground equipment to play safety. Two examples illustrate this. First, some experiences showed the importance of material choice in play equipment. It was said that wooden equipment holds the risk of splinters in your fingers. Further, it was experienced that the ropes of a horizontal climbing net were rather uncomfortable to hold. As a possible improvement, a girl suggested: “*At another playground they had something else (rubber handles) and when you climb, your hands don’t get hurt.*” *(School 2)*. Second, some children discussed that the risk for bad falls increases with increasing height of play frames, in particular for younger children. e.g., a boy in response to a girl’s wish to add a higher climbing frame: “*Yes, but what if those little children climb in the bigger ones? Then they fall from it.*” *(School 1).* A playground supervisor that is continuously present to watch over and help children in trouble and restricting use of high frames to older children were suggested as solutions. 

##### Safety of Playground Surroundings

Two of the discussed safety issues were related to the direct surroundings of the school playground: fence safety and traffic safety. At School 2, children evaluated the fence points of the medium-high fence that surrounded their playground as dangerous, since children had hurt themselves when they tried to climb over the fence. At School 3, children felt that fast driving cars near the playground created dangerous situations during recess. A girl explained about running after the ball: “*Yes, because then the ball rolls (on the road) and then… bam, along comes a car.*” *(School 3)*. Children explained that the current warning sign for car drivers had no effect and they suggested to move it to a more visible location and to add a pedestrian crossing. 

#### 3.1.3. Rules and Supervision

Children’s discussions on rules focused on both undesired and desired rules as well as on consistency of rules, transparency about rules and application of rules. Children indicated that they do not like to be restricted by rules in their freedom to play the way they want to play in the playground. At School 1, children expressed strong dissatisfaction about the high number of rules forbidding certain active play. For example, children indicated that it was not allowed to climb in trees, to run fast after one another, to stand on a play wall and to play on the stairs. They regarded their playground supervisors as over-concerned about their physical safety and considered this an important cause for many of the restrictions. A girl explained: “*They should let you do more. (…) It could be a nice playground if you could do more. There are some fun things, but you’re not allowed to play with them*”. In a mocking tone she considered the supervisors’ attitude: “*There should be zero risk that you fall or get hurt*.” *(School 1).* According to a rule at Schools 2 and 3, part of the playground was for younger and the remaining for older children. This separation was viewed as a loss of play opportunities: “*The playground for the younger children is lots of fun and you can do all kinds of things there, but we’re not allowed to go there*.” *(School 3).* Another boy said: “*Only young children are allowed, but older children also enjoy going on the slide.*” *(School 3)*. Children reported that they sometimes (secretly) violated rules. 

It appeared that children did not always recognize the arguments for specific rules and some expressed a wish to get to know these, for example: “*I think it’s stupid that when playing outside you are not allowed coming there. Why is that not allowed?*” *(girl, School 1)*. The same went for who is responsible for making up the rules, as illustrated by the following conversation. Girl 1: “*Does somebody set up the rules that are told by the supervisors?*” (…) Girl 2: “*Because then we could get angry at that person.*” Girl 1: “*And then we could make that person change these*.” *(School 1)*. Moreover, conversations showed that not all children understood the rules in the same way. At School 1, this could be explained by the researcher’s observation that playground supervisors often had their “own” rules. At School 3, there was a lack of clarity about soccer field reservations due to poor visibility of the schedule. 

Despite criticism on the number and content of rules, a recess in the playground without rules was not preferred either. At least this was the case at School 1, where rules were a major issue. At School 1, children were decisive that rules about social manners are desired and should be strictly maintained. These included: do not bully, kick and hit one another. Teasing is only allowed when not only the teaser but also the teased one feels it is humorous. Further, the reasonableness and consistency of rules were considered important. For example, children accepted the rule stating that playing soccer beyond the soccer field was not allowed because of safety issues: “*To be honest, I understand why it’s not allowed in the playground, because people can get hit on the head by a ball. If that would happen to me, I’d also think it wasn’t very nice.*” *(girl, School 1)*. However, playing alternative games with soft balls beyond the soccer field should not be forbidden. At School 3, children wanted rules for soccer (see “Peers”). On the researcher’s question how playground rules could be improved, children at School 1 suggested that they should be consulted about which rules would be good, and a suggestion box should be introduced where they can deliver ideas about rules. Of course, it would be necessary then that adults also act upon children’s input. Another rule that was discussed included the length of recess periods. Some children indicated that they wanted more recess time in order to have more play time in the playground. 

The enforcement of rules by playground supervisors was of great interest to children of School 1, since they were rather unhappy with the current situation. As summarized by a boy: “*The supervisors should be less strict, we think, because they get angry very easily and get tough.*” *(School 1)*. It was explained that rules were often too strictly maintained and not always communicated in a friendly way (tone and wording). Some nuancing in applying them was desired. For example, it was not allowed to throw hard (icy) snowballs during snow fights, yet supervisors considered almost every snowball hard. That children value nuancing was also seen at School 3. With a happy voice a girl explained about hanging on a basket: “*That’s actually not allowed, but it always makes our Sir laugh and he doesn’t forbid us*.” *(School 3).* Further, sanctions imposed on violating rules were felt to be too severe. For example, it was mentioned that supervisors often took their ball away. A boy suggested explaining the supervisors that this punishment strongly decreases children’s play opportunities, since a ball is necessary for many games. 

Children felt that their supervisors did not pay enough attention to maintaining rules about issues that really mattered to children. A girl explained: “*The rules that are really necessary (about social manners) are hardly paid attention to.*” *(School 1)*. They expected their supervisors to actively act upon unacceptable behaviour between children. More in general, children viewed a role for supervisors in helping children to solve arguments and to create a kind, peaceful and happy atmosphere in the playground. An active, friendly and caring attitude was considered an important characteristic of the ideal supervisor. A boy about his favourite supervisor: “*He’s really cool; sings the whole time, is an Ajax (Dutch soccer team) fan, giving everybody a high five. But the others scream like “no, no, don’t do that”.*” *(School 1)*. Unfortunately, the ideal attitude was not often seen among their current supervisors, according to the children. A playground with many supervisors was not preferred, in particular not when they behave passively and uninterested. 

#### 3.1.4. Peers

Children reported that play fellows are important for engaging in fun and active play in the playground. Conversations showed a desire to play with others, friends often being preferred over random children, in contrast to playing alone. Group games, both traditional and self-invented ones, were much liked. The presence of others was related to play opportunities in the playground. A girl explained that: “*When you’re alone in the playground, when your friends are ill, then there’s hardly anything to do*”. Another girl added: “*Well, actually, nothing at all… except for walking on stilts*.” *(School 1)*.

On the other hand, it was experienced that others could frustrate play activities as well, as a result of which play opportunities could not always be used or enjoyed to their full extent. Children talked about several events during which other children disrupted their play on purpose. One story illustrated that regular unfriendly or unpleasant behaviour of boys towards girls during soccer (e.g., not passing the ball to girls, verbal aggression) resulted in girls becoming less active than they wanted. A girl explained: “*We don’t like the way the boys react sometimes. Not all boys behave like that, but sometimes they do, some boys. And then we just pass the ball to them and don’t try it ourselves.* *We don’t dare to, to really do our best.*” *(School 3)*. It was suggested to make a sign stating that boys and girls are equal, in addition to a schedule indicating that boys and girls should alternate possession of the ball. Further, it was mentioned that some older children were not so kind to younger children. For example, children shared experiences about older children who pushed them aside, did not give them a turn in games and said unfriendly things. A boy reported similar events about the behaviour of friends *vs.* non-friends. Fights and angry reactions of others were felt to make playing in the playground less fun. Illustrative is an account of a girl who explained why a specific (non-school) playground visit from the past was fun: “*The nice thing was that everybody was together. There weren’t even any arguments.*” *(School 3)*.

### 3.2. Keeping the Playground Fun

Children explained that after a while playing in the playground can become boring due to repetition and a lack of variation in activities. In a conversation with the researcher in the playground, a girl said: “*Look, those children are skipping. But when you do that for a very long time, it becomes boring. Don’t you ever have that? That when you do something so often, it becomes boring?*” *(School 3)*. Children viewed a relationship between boredom of the playground and their age group. The following account is illustrative: “*Group 4 sees it (the playground) as normal, but it’s starting to get boring for them. For group 3, the playground’s really fun, because they’re allowed to play there now and to them it’s still new.* *But to us (group 7), it’s still pretty old.*” *(boy, School 3)*. At a certain moment all play opportunities have been used over and over again and then children can get tired of the playground. However, some children hold the opinion that playing soccer always remains fun. 

At School 1, children suggested introducing new equipment to overcome this problem. However, as a girl described: “*When something new is put down you eventually become fed up of that as well.*” *(School 1)*. A similar solution was proposed at School 3, but instead of static equipment a boy suggested to choose for flexible equipment. He showed the others a system that was made up of different play elements, such as tubes, steps, slides and walls, which could be assembled in multiple ways. As he explained: “*Then it (play equipment) changes continually and then it becomes fun again*.” *(School 3).* The others agreed and were very enthusiastic about it. Lastly, it was suggested to search for new playground games at the Internet, so that the same playground could be approached in a new and fresh way. 

### 3.3. Project Evaluation by Participants

Children mentioned different reasons for participating in the study. For example, they thought the project would be interesting and fun (13/18 children), they wanted to improve their playground (5/18) or considered their playground boring (2/18). The majority of the children felt that the playground could be improved (yes: 15/18, maybe: 3/18). At the end of the project, all children found the project to be fun and interesting. Most felt they were experts in playgrounds (yes: 5/18, a little: 8/18, no: 5/18). At School 3, the following conversation developed about children as experts. Girl 1: “*They (children) play outside a lot*”. Researcher: “*What do you mean*”? Girl 1: “*Children play in the playground, we know what they want*”. Girl 2 added: “*We understand it better*”. Girl 1 commented: “*Adults have really boring ideas*”. Most children stated that they felt at ease during the project (yes: 14/18, a little: 3/18, 1 missing), were able to express their opinion (yes: 14/18, a little: 4/18) and that something was done with their recommendations (yes: 13/18, a little: 2/18, no: 3/18). They also mentioned a range of learning outcomes, including what children consider a good playground, how to collaborate (School 1), form your opinion about the playground, not be nervous at presentations, create ideas (School 2), that playgrounds can be boring, how to change the playground and why, how to make a film (School 3) and how research works (Schools 1 and 3). Two children reported that they learned nothing, whereas three children did not answer the question on learning outcomes. In addition, children expressed the hope that adults would learn how children think about playgrounds and how playgrounds can be improved (all schools) and that children know a lot, have an opinion and that adults can listen to it (School 1). 

## 4. Discussion

Understanding the child-perspective of school playgrounds is necessary to gain a deeper understanding of determinants of children’s playground PA. This study explored 9–12 years old primary schoolchildren’s perceptions of their school playground, using a participatory research design that gave children a voice as co-researchers. The results suggest that children have a desire to use the playground as a PA or “active play” environment during recess, as long as the available play opportunities allow playing to be fun. Otherwise, children tend to engage in sedentary activities. Children identified physical characteristics, perceived physical safety, rules and supervision, and social interactions with peers as important domains of determinants of play opportunities for fun and active playground play. To keep a playground attractive over time, variation in and renewal of equipment and games were recommended. This is especially relevant to older children, since they already played for years in the playground. 

Our finding that fun is an important aspect of active play is in line with previous work reporting that children characterize play as fun, along with descriptions such as spontaneous, energizing, preventing boredom, interactions with friends, less structured and freedom [40,41,42]. Enjoyment of PA is thought to lead to being intrinsically motivated to engage in the activity and to be associated with positive affective feelings [43]. It has been identified as a correlate of PA in children and adolescents [44,45]. Our results suggest that for fun and active play children need an enjoyable playground environment. This idea is supported by the positive association of children’s liking of the primary school play environment with children’s PA during recess [46]. Given our results and those of others, we hypothesize that children’s liking of the playground environment is an important indicator of its activity-friendliness.

The present study showed that children relate the amount of play space to opportunities for fun and active playground play, thereby supporting previous qualitative studies [19,20,21,42,47]. However, quantitative research showed no association between playground size and children’s playground PA [11]. A possible explanation is that the objective playground size is not equivalent to the child-perceived amount of play space. In our study, children indicated that the latter was decreased by factors such as the presence of other children and impractical placement of equipment or bikes. Indeed, decreased playground density (m^2^/child) was found to be associated with higher playground PA [11]. King *et al.* [48] found that when children’s play space was reduced, their perception of choice in their play reduced as well. Hence, perceived level of choice may partly explain the relation between perceived play space and playground PA.

The importance of sufficient and accessible play equipment for playground PA has been reported previously (e.g., [11,12,18,19,21,22]). Our study added insight into children’s desires regarding the composition of the equipment. A combination of both fixed and loose equipment that is varied and challenging for both young and older children and suitable (at least some materials) for multiplayer play was preferred. Materials that did not have an obvious play function and “green” equipment such as vegetation were enjoyed and desired for play as well. Similarly, Powell *et al.* [22] demonstrated that children also use imaginative play to manipulate fixed equipment for new play purposes. We found that children’s enjoyment of individual play materials and the suitability of equipment for play were influenced by characteristics like function, design and material. This suggests that not only the presence but also the child-perceived quality of play equipment is important for activity-friendly playgrounds. Since characteristics of individual play materials have not been subject to many PA research yet, more investigation on this topic is recommended.

The results also showed that children prefer to play in a clean playground. Previously, children described the absence of litter as a characteristic of nice playgrounds [18]. Nevertheless no association with children’s observed PA level was found [49]. However, cleanliness was defined as visible dirt and evaluated by academic researchers. In our study, children emphasized the problem of urine, which is easily overlooked by others than the primary users of playgrounds. Further, our results suggest that aesthetic elements like colour and greenery contribute to a positive atmosphere in the playground. Teachers also argued that playground aesthetics encourage children’s active play [18]. 

Another notable finding was children’s awareness of and concern about unsafe playground conditions with regard to playground surfacing, play equipment and immediate surroundings of the playground. Children aimed for a playground environment that reduced the risk of injury, *i.e.*, high physical safety, but nevertheless allowed them to create their own active and challenging play. Colabianchi *et al.* [49] found no association between researcher-assessed safety, defined in terms of equipment height and presence of soft landings and railings, with playground PA outside school hours. However, given our results, the used conceptualization of physical safety might be too narrow. Another study looked into children’s perceptions of school safety in relation to PA, but used no playground-specific safety and PA measures [50]. In a qualitative study children reported injury avoidance as a barrier for playground PA [19]. More research is needed to understand children’s ideas of physical playground safety and its association with PA.

Consistent with previous qualitative studies [18,19,51], we found that children regularly experienced adult-imposed rules as a barrier for active play, limiting their possibilities to use equipment and create their own play activities. Some rules were viewed as unwanted and unnecessary efforts of supervisors to reduce the physical safety risks of play. Children consider risk a pleasurable aspect of play that is linked to overcoming challenges and mastering activities [19,52]. An overemphasis of schools on safety by promoting structured play may thereby reduce children’s motivation and opportunities for PA in the playground [51,52]. Nevertheless, the present study also indicates that children prefer to have a minimum number of clearly defined rules to support a socially and physically safe playground environment. These rules are preferably set up in collaboration with children to contribute to their appreciation of and acting upon the rules. Children indicated that supervisors should apply the rules with some flexibility. The latter enhances their play choices, thereby supporting the play process [48]. 

Peers may positively or negatively affect playground PA levels of children. Playmates—especially friends—were considered facilitators of active play, as was described previously [17,19,20,22,42]. Many preferred play activities were done together or were typical group games, involving physical and social interactions with peers. From a developmental perspective, it has been suggested that friendships increasingly become important with age [53] and previous studies among youth have shown that the presence of peers and friends may increase the likelihood of engaging in PA [54]. Nevertheless, peers also appeared to negatively influence active play. Previously, boy dominance, conflicts and bulling were identified as barriers to recess PA [19,20]. We found socially and physically aggressive behaviour, bullying, peer pressure and dominance between genders, (non-)friends and age groups to have an impact. Importantly, the present study showed that children view a role for both supervisors and themselves to stimulate a positive climate in the playground. 

This study has several limitations. First, at School 1 the group meetings were more structured by the academic researcher than at Schools 2 and 3. However, as indicated in the process evaluation, we believe that children were not limited in their freedom to express their perspectives and introduce new issues. Moreover, the researcher was aware of the increasing relationship of trust, among others indicated by some children developing from shy towards more expressing and self-confident. Second, our results may be limited in generalizability as only motivated children participated. However, we experienced that nearly all of the approached children were enthusiastic and willing to participate, including both shy and reserved children as well as more assertive children. In addition, though only a small number of children per school could participate in the meetings, the findings were well-received by their classmates (Schools 2 and 3). Hence, we believe the results are representative for the participating schools. Third, there is the issue of generalizability of the results for primary schools in general. Participatory research is by definition locally situated, meaning that the generated knowledge is context-specific and the findings should be interpreted accordingly. Nonetheless, comparing local projects (*i.e.*, schools) enables the identification of general patterns that can be transferred when you understand the contextual conditions of both the original settings and the new setting [31]. We found most of the categories emerging from the group meetings to be similar across schools (Table 2), implying that the identified determinants might be relevant to other schools as well. A fourth limitation is that we included children from three schools in Amsterdam. It would be interesting to repeat this study format at more schools in a wider geographic area, to increase the robustness of our results. Finally, all schools had an interest in using children’s suggestions to improve the school playgrounds, however, improvements were not made during the course of this study. Though a previous study demonstrated that active play significantly contributes to primary schoolchildren’s daily PA levels [55], future studies should explore whether the suggested improvements of the children actually increase their MVPA in the school playgrounds.

Strengths of this study include the child-centered, participatory design that allowed children to freely express themselves and to collectively give meaning to the concept of activity-friendly playgrounds. In addition, using multiple participatory activities enabled children to share their perceptions in a way that fitted their interests, skills and competencies. Furthermore, working with children in multiple follow-up meetings helped to create a relationship of trust and provided the opportunity to deepen and verify perspectives, resulting in detailed data.

## 5. Conclusions

This is the first participatory study of 9–12 years old children’s perspectives of determinants of activity-friendly school playgrounds, by inviting them as co-researchers. Our findings are an important contribution to both the literature and practice as they provide concrete, *i.e.*, context-specific, starting points towards positive changes in the lives of children. Children indicated that the activity-friendliness of school playgrounds is influenced by characteristics of the playground itself as well as the social situation in the playground. For fun and active play, it is important that children (1) can do a variety of activities (equipment, maintenance, aesthetics, playmates); (2) are allowed to do a variety of activities (rules, supervision); and (3) can do these activities safely (physically and socially). What matters is how children themselves experience these conditions. Familiarity with the playground increases as children grow older, making variation in equipment and games more important. This implicates that playgrounds should have a flexible design to remain attractive for physical activity for all school-aged children. 

Our findings highlight the importance of an integrated and multi-facetted approach to developing activity-friendly playgrounds that stimulate children to be physically active during recess. A physically attractive playground with lots of play equipment is not sufficient to get children active. Children should be able to use and enjoy these play opportunities, which asks for a perceived physically safe and supportive social playground environment as well. 

We view an important role for children in the development of activity-friendly playgrounds. Our research shows that, as the primary users, children know the playground environment inside out and are able to identify barriers for active play that are easily overlooked, unknown or differently-perceived by adults. An important characteristic of participatory research is that it is context-specific implying that no “one size fits all school playground” exists. Our study identified important playground themes that should be evaluated in different playground contexts. We recommend schools to invite their pupils to express how they perceive these items in their own school playground. Future studies are needed to examine whether perspectives of activity-friendly school playgrounds differ between boys and girls, children of different age or children with different socio-economic backgrounds. 

We experienced that children are strongly motivated to express their views, especially when the research process involves play. Moreover, children come up with concrete solutions to problems and are aware of advantages and disadvantages of their ideas. Hence, we believe that more structural involvement of children in designing, developing and improving playground environments may increase the opportunities for fun and active play of these environments and consequently children’s PA levels during recess. 

## Figures and Tables

**Table 1:** Neighbourhood, school, playground, recess and participant characteristics per school.

Characteristic	School 1	School 2	School 3
**Neighbourhood**			
Socioeconomic status (2010)	High	Low	Medium
Percentage of 10-year old children who are overweight **^a^**/obese (2013/2014) **^b^**	7.2/0	26.9/6.1	21.6/5.2
**School**			
Educational approach	Montessori	Ecumenical	Dalton Plan
Number of pupils (2013/2014)	300–400	100–200	300–400
**Playground ^c^**			
Size (m^2^) **^d^**	≈1700	Section (I) **^e^** ≈ 400 + Section (II) **^f^** ≈ 3800	≈2500
Field(s) and (single) goal(s)	1 soccer/basketball field with 2/2 goals, 1 single basketball goal, 1 single soccer goal	(I): none (II): 1 soccer/basketball field with 2/2 goals	1 soccer/basketball field with 2/2 goals, 1 single basketball goal
Fixed equipment	2 table tennis tables, 1 climbing frame, 4 tumble bars, 1 paint mark, 2 play hills, multiple sitting rocks	(I): 1 play frame, 1 sandpit, 1 bench (II): 1 table tennis table, 3 play frames, 4 swings, 2 roundabouts, 1 sandpit, 1 paint mark, 1 play hill, 1 seesaw, 2 spinning attributes, multiple benches	3 play frames, 1 climbing frame, 7 tumble bars, 4 swings, 3 roundabouts, 1 sandpit, 2 paint marks, multiple benches
Surface type(s)	Paving stone, rubber paving	Asphalt, paving stone, rubber paving	Asphalt, paving stone, rubber paving, gravel
Nature	Some trees	Some trees	Some trees
**Recess**			
Length	30 min	Recess (1): 15 min Recess (2): 15 min	Recess (1): 15 min Recess (2): 40 min
Number of groups at playground	8 groups	3 groups	(1): 5–6 groups, (2): 10 groups
Supervised by	Seniors of an external organisation	Teachers	(1): teachers, (2): parents
**Participants**			
Gender	4F/2M	3F/3M	3F/3M
Age range (years)	9–11	10–11	10–12
Relation	From 3 classes, but familiar with each other	From 1 class	From 1 class

Table legend: **^a^** Overweight including obesity. **^b^** Obtained from the Public Health Service of Amsterdam [35] and defined according to the Cole criteria [36]. **^c^** Independently assessed in May and June 2014 by two researchers. **^d^** Calculated using Google Maps. **^e^** School property. **^f^** Owned by municipality.

**Table 2:** Coverage of categories across schools.

Theme	Emerged at		
	School 1	School 2	School 3
Category			
**Play**			
Activity and relaxation	x	x	x
Fun	x	x	x
Boredom	x	x	x
Games	x	x	x
Variation *vs.* repetition	x		x
**Physical Characteristics**			
Size and free space	x	x	x
Fixed play equipment	x	x	x
Loose play equipment	x	x	x
Alternative play equipment	x		
Amount of play equipment	x	x	x
Variation in equipment	x	x	x
Accessibility of equipment	x		x
Level of difficulty of equipment	x	x	
Suitability of equipment for play	x	x	x
Quality of equipment		x	
State of maintenance	x	x	
Aesthetics	x	x	
**Physical Safety**			
Surface safety	x	x	x
Equipment safety	x	x	x
Safety of playground surroundings		x	x
**Rules and Supervision**			
Undesired rules	x	x	x
Desired rules	x		x
Consistency of rules	x		
Transparency about rules	x		x
Application of rules	x		x
Nature of supervision	x		
**Peers**			
Playing together	x	x	x
Negative behaviour of peers	x	x	x

## Abbreviations

The following abbreviations are used in this manuscript:

PA	physical activity
MVPA	moderate-to-vigorous physical activity
